# A pathophysiological intersection between metabolic biomarkers and memory: a longitudinal study in the STZ-induced diabetic mouse model

**DOI:** 10.3389/fphys.2025.1455434

**Published:** 2025-03-12

**Authors:** Maria Teresa Venuti, Elisa Roda, Federico Brandalise, Meghma Sarkar, Mattia Cappelletti, Attilio F. Speciani, Irene Soffientini, Erica Cecilia Priori, Francesca Giammello, Daniela Ratto, Carlo A. Locatelli, Paola Rossi

**Affiliations:** ^1^ Department of Biology and Biotechnology “L. Spallanzani”, University of Pavia, Pavia, Italy; ^2^ Laboratory of Clinical and Experimental Toxicology, Pavia Poison Centre, National Toxicology Information Centre, Toxicology Unit, Istituti Clinici Scientifici Maugeri IRCCS, Pavia, Italy; ^3^ Department of Biomedical Sciences, Div. Neuroscience and Clinical Pharmacology, University of Cagliari, Cagliari, Italy; ^4^ GEK Lab, Milano, Italy

**Keywords:** diabetes, MGO, glycated albumin, recognition memory, hippocampus, pancreas, streptozotocin-induced

## Abstract

Diabetes mellitus (DM) is a metabolic disorder characterized by high blood sugar levels due to insufficient insulin production or insulin resistance. Recently, metabolic biomarkers, such as glycated albumin (GA) and methylglyoxal (MGO), have been successfully employed for the management of diabetes and its complications. The main goal of this study was to investigate the relationship between metabolic parameters, related to diabetic conditions, and the recognition memory, a declarative episodic long-term memory, in a streptozotocin (STZ)-induced diabetes mouse model. The longitudinal experimental plan scheduled five experimental timepoints, starting from 9 months and lasting until 19 months of age, and included different evaluations: i) fasting serum glucose, GA, and MGO, ii) recognition memory performance; iii) histological examinations of pancreas and hippocampus. At 13 months of age, mice were randomly divided into two groups, and STZ (50 mg/kg i.p.) or vehicle was administered for 5 consecutive days. Mice were fed with a normal diet but, starting from 14 months, half of them were given water with a high sugar (HS) to explore the potential detrimental effects of HS intake to hyperglycemia. Our main outcomes are as follows: i) HS intake alone does not contribute to worsened diabetic condition/hyperglycemia; ii) GA emerges as a reliable biomarker for monitoring diabetic conditions, consistently increasing with hyperglycemia; iii) diabetic conditions correlate with a worsening of recognition memory; iv) diabetic mice display mild-to-severe insulitis and injured hippocampal cytoarchitecture, detectable in Ammon’s horns regions CA1 and CA3; v) correlation among recovered normal fasting glycemic level and recognition memory, partial regaining of physiological pancreatic morphology, and hippocampal cytoarchitecture.

## 1 Introduction

Diabetes mellitus (DM) is a metabolic condition leading to chronic high blood sugar levels due to insufficient insulin production or ineffective effects of insulin on target cells. Two primary forms of diabetes have been characterized. Type 1 diabetes (T1DM) is typically diagnosed during childhood and is a multifactorial disease with a strong genetic component in which the pancreatic cells stop producing insulin. Type 2 diabetes (T2DM) is generally diagnosed in adulthood and is instead related to lifestyle factors. Both types result in long-term high blood sugar levels. T2DM is less severe, but it accounts for 90% of diabetes cases. It does not always require insulin for treatment and, therefore, is called an “insulin-independent” form of diabetes. The condition is primarily caused by long-term consumption of a high-calorie, high-sugar diet, which results in frequent and significant insulin spikes that eventually desensitize target cells, causing prolonged hyperglycemia (Guthrie and Guthrie, 2004; Nowotny et al., 2015).

One significant consequence of prolonged hyperglycemia is the increased formation of advanced glycation end products (AGEs), which activate AGE receptors (RAGE), leading to oxidative stress and increase in inflammation (Bierhaus et al., 2001; Soares et al., 2013; Meireles et al., 2015; Bettiga et al., 2019; Mori et al., 2024; Oliveira et al., 2024). This AGE–RAGE pathway is implicated in the development of diabetes-related complications such as cardiovascular disease, kidney damage, and neurodegenerative disorders (Giacco and Brownlee, 2010; Srikanth et al., 2011). DM is diagnosed and classified using blood biomarkers such as glucose and glycated hemoglobin (HbA1c), measured through various tests (Ramachandra Bhat et al., 2019). Monitoring these biomarkers is critical for preventing or managing the adverse physiological effects of diabetes (Yazdanpanah et al., 2017), and other emerging biomarkers like glycated albumin (GA) and methylglyoxal (MGO) are gaining attention (Kouzuma et al., 2002; Freitas et al., 2017; Piuri et al., 2020; Belinskaia et al., 2021).

GA is linked to organ damage, including kidney disease and atherosclerosis, and promotes insulin resistance through reactive oxygen species (ROS) generation (Wu et al., 2016; Paradela-Dobarro et al., 2019). It offers a viable alternative to HbA1c for assessing glycemic balance over shorter time periods, particularly when HbA1c cannot be measured (Freitas et al., 2017; Krhač and Lovrenčić, 2019). MGO, a reactive byproduct of glucose metabolism, serves as a precursor to AGEs and plays a pivotal role in the development of diabetic complications. Its accumulation contributes to vascular dysfunction, insulin signaling disruption, and chronic inflammation (Kong et al., 2020; Bhattacharya et al., 2023). High levels of MGO have been detected in diabetic patients (in plasma, pancreas, muscle, and adipose tissue) and correlated to a reduction in glucose and glutathione transporters. These disruptions impair vascular health and exacerbate diabetes-related complications such as chronic kidney disease and cognitive deficits (Ogawa et al., 2010; Li et al., 2012; Cai et al., 2014; A; Shamsaldeen et al., 2016; Gill et al., 2019; Ramachandra Bhat et al., 2019; Sutkowska et al., 2023). The role of AGEs in diabetes-induced cognitive decline is still unknown (Momeni et al., 2021).

Under physiological circumstances, MGO is detoxified by the glyoxalase system, which consists of glyoxalase I and II (Glo1 and Glo2), preventing excessive formation of AGE. The biochemical pathways through which MGO is linked to the development of diabetes, vascular complications of diabetes, and other age-related diseases have been described (Schalkwijk and Stehouwer, 2020).

Several epidemiological studies show that diabetes is the major risk factor for neurodegeneration: approximately 29% of people with T2DM experience cognitive decline and neurodegeneration. This association underscores the systemic nature of diabetes, which not only affects peripheral organs, but it also has profound effects on the central nervous system. Insufficient insulin uptake appears to be the main trigger for neurodegenerative mechanisms, particularly in the hippocampus, where insulin resistance induces cognitive decline associated with neurodegeneration (Shieh et al., 2020). It has been found that diabetes can speed up the progression from mild cognitive impairment (MCI) to severe dementia (Patel et al., 2016; Farajpour et al., 2017; Dove et al., 2021; Ding et al., 2024). Recognition memory, a declarative long-term episodic form of memory, is one of the most important features of human personality that is lost owing to MCI. A decline in recognition memory is observed in rodents with diabetes using various cognitive tasks, such as novel object recognition (NOR) or placement tests (Patel et al., 2016; Farajpour et al., 2017; Kassab et al., 2019; Presta et al., 2024). Cognitive decline is defined as a deterioration in cognitive functions, including difficulty with language and memory loss. Over time, cognitive decline can lead to disorders such as MCI or senile dementia. Specifically, one of the most studied diseases concerning glucose intake is Alzheimer’s disease (AD), which some authors define also as type 3 diabetes, for its suggested metabolic component (Yazdanpanah et al., 2017; Nguyen et al., 2020; Michailidis et al., 2022). The hippocampus plays a central role in memory and learning, making it a critical area affected in both dementia and Alzheimer’s disease (Rao et al., 2022). Hyperglycemia induces oxidative stress, inflammation, and neuronal damage, particularly impacting the hippocampus, a key region for memory and learning. Insulin resistance in the brain further disrupts hippocampal-dependent memory processes, contributing to cognitive decline as observed in MCI, dementia, and Alzheimer’s disease. Recognition memory deficits, commonly assessed through the novel object recognition (NOR) task, serve as a marker of early cognitive changes (Hu et al., 2019; Li et al., 2020; Barone et al., 2021; Aderinto et al., 2023).

High sucrose consumption has been shown to induce glucose intolerance, hyperinsulinemia, and hyperglycemia in different animal models (Oliveira et al., 2014; Flister et al., 2018; Melo et al., 2019; Seshadri et al., 2019). Alterations in metabolic profiles (hypertriglyceridemia and hyperinsulinemia) as well as increased liver lipogenic gene expression, oxidative stress, and inflammatory markers were evinced in rats fed for less than 21 days with 10% sucrose in their drinking water (Castro et al., 2024). The metabolic disturbances caused by high sugar intake, such as hypertriglyceridemia and oxidative stress, are paralleled by neuroinflammation and cognitive decline, suggesting that diet plays a pivotal role in the interplay between diabetes and neurodegenerative diseases (Coirini et al., 2022). Diets rich in refined sugar not only contribute to obesity but also impair cognitive functions like memory and cognitive flexibility (Kalaria et al., 2008; Kanoski and Davidson, 2011; Cheke et al., 2016). In particular, the effect involved hippocampal recognition memory performance in rodents, particularly during the early stages of cognitive decline (Beilharz et al., 2016). Furthermore, consumption of 35% sucrose-sweetened water for 9 weeks can lead to pre-diabetes and glucose intolerance in mice (Burgeiro et al., 2017). In a transgenic mouse model of AD, sucrose intake has been shown to exacerbate insulin resistance and amyloidosis, both of which contribute to memory deficits (Cao et al., 2007; Orr et al., 2014).

The antibiotic streptozotocin (STZ) selectively destroys pancreatic β-cells, which induces diabetes, thus providing valuable insights into the pathophysiology of diabetes. In particular, a high-dose STZ protocol induces the complete destruction of pancreatic β-cells, reproducing T1DM. On the contrary, a low dose of STZ induces a gradual death of pancreatic β-cells, mimicking the pathogenesis of T2DM (Hahn et al., 2020; Furman, 2021; Bauer et al., 2023a). These models have allowed scientists to explore the molecular mechanisms underlying diabetes-related complications, especially the role of hyperglycemia in disease progression. The STZ-induced diabetes model was chosen to monitor the progression of DM over time, with the goal of identifying new biomarkers involved in the onset and diagnosis of the disease. A preclinical animal model of diabetes was developed, opening the question of a long-term induction of type II diabetes with multiple low doses of STZ.

This study focuses on several key points: the potential use of new biomarkers for T2DM, such as GA and MGO, and the relationship between those peripheric metabolic biomarkers and cognitive functions. Specifically, we explore how these biomarkers are linked to cognitive performance, including declarative memory, focusing on recognition memory, a long-term form of memory. Furthermore, the experimental model has allowed us to assess the recovery in time of both peripheric metabolic biomarkers and cognitive performance, thus obtaining a confirmation of their direct relationship.

## 2 Material and Methods

### 2.1 Animals

Thirty-five 9-month-old wild-type male mice (strain C57BL-6J) were maintained on a 12-h light/dark cycle in single cages in the Animal Care Facility at the University of Pavia. Water and food were provided *ad libitum*. All experiments were carried out according to the guidelines laid out by the institution’s animal welfare committee, the Ethics Committee of Pavia University (Ministry of Health, License number 220/2022-PR), also in compliance with the European Council Directive 2010/63/EU on the care and use of laboratory animals.

Mice were fed the animal facility’s diet (normal diet or ND), which was a standard 4RF21 pellet supplied by Mucedola Srl. The pellet was prepared following the standard pelleting procedure, which involved using dry saturated steam, a drying phase, and subsequent cooling.

Table 1 shows the nutritional composition of the ND pellet.

**TABLE 1:** Nutritional composition of control diet pellets (ND). The energy value was 387.6 Kcal/100 g.

Composition	g/100 g
Carbohydrate	42.63
Sugar	3.68
Protein	18.50
Fat	3.00
Fiber	6.00

### 2.2 *In vivo* longitudinal study

**FIGURE 1:**
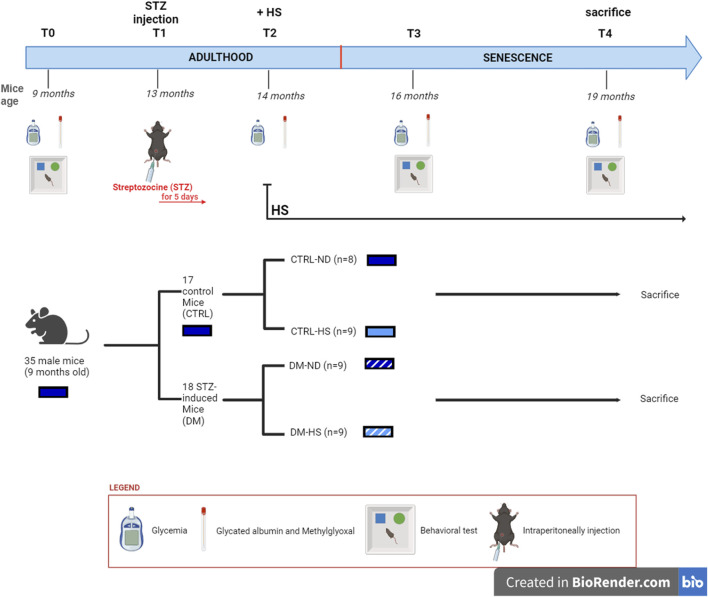
Experimental plan.

The investigational design scheduled five experimental timepoints (Figure 1), starting from 9 months (T0) and lasting until the mice reached 19 months of age. T0: at 9 months, all animals were fed the ND; fasting glycemia, glycated albumin (GA), and methylglyoxal (MGO) were measured, and spontaneous behavioral tests were performed. T1: 13-month-old mice were randomly divided into two groups: i) 17 control animals (CTRL) were intraperitoneally (i.p.) injected with physiological saline (0.9% sodium chloride) for 5 consecutive days, while ii) the other 18 mice (namely, DM) were i.p. injected with an STZ solution to induce diabetes (namely, STZ induction, see detailed protocols described in the following paragraph). One month after the injections (T2), fasting glycemia, GA, and MGO were assessed in all mice to verify the efficacy of STZ induction. Starting at T2, half of the mice belonging to each group, both CTRL and DM, were supplemented with 10% sucrose water *ad libitum* as their only source for drinking (high-sugar group or HS). Therefore, mice were divided into four groups: CTRL fed with ND (CTRL-ND), CTRL watered with HS (CTRL-HS), DM fed with ND (DM-ND), and DM watered with HS (DM-HS). After 16 months (T3) and 19 months (T4), fasting glycemia, GA, and MGO were monitored, and spontaneous behavioral tests were performed. All animals were sacrificed at the last experimental timepoint (T4), and organs were collected as described (see section *Material and Methods* Section 2.6).

### 2.3 Diabetes induction

Diabetes was chemically induced by intraperitoneal injection of STZ for 5 consecutive days, according to Animals Models of Diabetic Complication Consortium (AMDCC) (Wu and Huan, 2008; Furman, 2021). Most papers reported diabetes induction performed at murine ages ranging from 8 to 12 weeks (3 months), equivalent to approximately 20 human years, which corresponds to the early/juvenile adulthood phase in humans (Holstad and Sandler, 1999; Nørgaard et al., 2020; Furman, 2021). Differently, in our current investigation, diabetes has been induced at 13 months of age (T1), approximately equivalent to 43 human years, corresponding to the late adulthood phase. We specifically selected this age, which corresponds to the late adulthood phase, to mimic the pathology of human type 2 diabetes and its distinctive onset, which typically develops in adulthood at the age of 45 years (Reed et al., 2021; Carrillo-Larco et al., 2024). It should also be taken into careful consideration that the choice of diabetes induction at 13 months of age allowed us to minimize the impact of synaptic plasticity phenomena associated with recognition memory during the juvenile phase.

In brief, to induce diabetes, a 50-mg/kg STZ dose, dissolved in 100 mM citrate buffer solution, was administered daily by i.p. injection to mice belonging to the DM group (namely, STZ mice), while control animals received physiological saline (0.9% NaCl). At T2, 1 month after the last treatment, blood glucose was measured by tail vein sampling, both in CTRL and STZ animals which have been fasted for 4 h. The STZ mouse was considered diabetic if the fasting blood glucose exceeds 300 mg/dL for three consecutive measurements. In the mouse model, fasting blood glucose level exceeding 300 mg/dL up to 600 mg/dL (16.7 mmol/L to 33.3 mmol/L) is considered a criterion for classification of diabetes (Graham et al., 2011; Furman, 2021).

### 2.4 Measurement of fasting glycemia, GA, and MGO

Mice were fasted for 12–16 h before fasting glycemia, GA, and MGO measurements. Blood was collected from the tail vein. Specifically, a drop of blood was taken to assess blood glucose, run on a reactive strip, and then read using a OneTouch Verio Reflect® glucometer (Lifescan Italy Srl).

To assess fasting blood sample collection to measure serum GA and MGO, 200 µL of blood was collected using synthetic swabs and analyzed by the GEK Lab laboratory using a specific ELISA kit. In particular, the human GA concentrations in plasma samples were determined using a Mouse Glycated Albumin (GA) ELISA Kit (LLD 11.3 pmol/mL, Abbexa Ltd., Cambridge Science Park, Cambridge, U.K.), and the MGO was determined using the OxiSelect™ Methylglyoxal Competitive ELISA Kit (lower range of detection 0 μg/mL, Cell Biolabs, San Diego, CA, USA), which is an enzyme immunoassay developed for the detection/quantitation of protein adducts of methylglyoxal-derived hydroimidazolone (MG-H1) (Nowotny et al., 2015).

### 2.5 Behavioral test and cognitive frailty index

A spontaneous behavioral test was performed to assess murine recognition memory. In particular, mouse activity was quantified by a SMART video tracking system with a selected sampling time of 40 ms/point (2Biological Instruments, Besozzo, Varese, Italy) and a Sony CCD color video camera (PAL). At selected timepoints, i.e., T0, T3, and T4, all mice performed the novel object Recognition (NOR) task, which is a highly validated test for assessing recognition memory, to evaluate their ability to recognize a novel object in the environment, hence assessing the efficacy of their memory for the objects.

#### 2.5.1 Novel object recognition (NOR) test

The novel object recognition task was carried out as previously described (Brandalise et al., 2017; Ratto et al., 2019; Roda et al., 2022); in particular, the procedure consisted of three primary phases: open arena, familiarization, and test. During the test phase, the number of approaches and the time of approaches to the familiar and the novel objects were measured as cognitive parameters. To evaluate the discrimination between novel and familiar objects, the mean novelty discrimination index (DI) was calculated by using the following formula (Silvers et al., 2007):

$$DI=\left(n-f\right)/\left(n+f\right)$$

where (n) is the average time or number of approaches to the novel object and (f) is the average time or number of approaches to the familiar object (Table 1). The DI ranged from −1 to 1, where −1 means a complete preference for the familiar object, 0 means no preference, and 1 means a complete preference for the novel object.

For each parameter, the corresponding locomotor frailty index (FI) was calculated by using the following formula (Parks et al., 2012):

$$FI=\left(Value-Mean\;\;Value\;\;at\;\;T0\right)/\left(SD\;\;at\;\;T0\right)*0.25.$$

### 2.6 Morphological evaluation of the pancreas and hippocampus

At T4, all mice were deeply anesthetized before decapitation using isoflurane inhalation (Aldrich, Milwaukee, WI, USA). The head/body of the pancreas and the brain were immediately excised, washed in 0.9% NaCl, fixed by immersion for 7  and 48 h at room temperature in 4% paraformaldehyde in 0.1 M phosphate buffer (pH 7.4), and post-fixed in the same fixative medium at 4°C for 1.5 h. Then, tissues were kept in absolute ethanol for 1 h, followed by acetone, and finally embedded in Paraplast X-TRA (Sigma-Aldrich, Milan, Italy). Using a manual rotatory microtome, 6-µm-thick pancreatic and 8-µm-thick brain coronal sections were cut serially and collected on silane-coated slides.

#### 2.6.1 Light microscopy: H&E

To reveal the pathophysiology and estimate potential structural pancreatic and hippocampus alterations, hematoxylin and eosin (H&E) staining was performed as previously described (Kádár et al., 2009; Lattouf et al., 2014; Roda et al., 2017; Luca et al., 2023).

In particular, since both the pancreas and CNS possess more complex specialized structures compared to other tissues, the H&E allows for an overview of tissue structures, anatomical order, and area-specific settings (Jordan et al., 2011; Zhang and Xiong, 2014; Roda et al., 2019; Longnecker, 2021; Li et al., 2022). Therefore, using the brightfield examination of H&E-stained samples at low magnification, pancreatic and hippocampal site identification was achieved, allowing the identification of precise brain sections and also distinguishing typical pancreatic microanatomy. In particular, concerning the pancreas, the coronal aspect was determined by examining the morphology of pancreatic ducts and islets of Langerhans. Specifically, in case of a spherical-shaped duct lined with the cuboidal epithelium, the coronal plane orientation was assumed.

Sections were observed using a Leica DM6B WF microscope (Leica Microsystems, Buccinasco, MI, Italy). The images were acquired using a Leica DFC 7000 t CCD camera (Leica Microsystems, Buccinasco, MI, Italy) and stored on a PC running the Leica Application Suite X (LAS X) software (version 5.1.0). The imaging system (LAS X Navigator) and the merge function were used to reconstruct the whole hippocampus.

##### 2.6.1.1 Insulitis scoring

H&E-stained pancreatic tissue sections were then observed for the assessment of lymphocytic infiltrates in the pancreatic islets of Langerhans. A minimum of 30 islets/group were scored for insulitis. Scoring was performed under double-blinded conditions. The degree of insulitis was graded according to the following criteria: normal islet, score 0; perivascular/periductal infiltration, score 1; peri-insulitis, score 2; mild insulitis (<25% of the islets infiltrated), score 3; and severe insulitis (more than 25% of the islets infiltrated), score 4 (Pejnovic et al., 2013; Pavlovic et al., 2018).

##### 2.6.1.2 Hippocampal injury evaluation

For histopathological evaluation, five slides (approximately 20 sections) per mouse were examined. The most representative figures of the hippocampus were selected and are shown. In particular, the dentate gyrus (DG) and Ammon’s horn region were scrutinized. Concerning the quantitative evaluation, the following were measured: i) whole thickness of the DG layer; ii) pyramidal cell layer thickness of CA subdivisions; iii) cell density (number of cells/area in mm2).

### 2.7 Statistics

The values obtained were expressed in terms of the mean ± standard error of the mean (SEM, standard error of the mean). The statistical analysis of Kaplan–Meier graphs was obtained with a log-rank (Mantel–Cox) test. To evaluate the statistical differences between different experimental groups in fasting glycemia, GA, MGO, weight, global DI, global FI, and quantitative analysis of hippocampus tissue, the one-way ANOVA test was used, followed by the Bonferroni *post hoc* test. The repeated measures ANOVA test followed by the Bonferroni *post hoc* test was carried out for the comparison of glycemia, GA, and MGO in CTRL and DM experimental groups. Microsoft Excel and Prism 9 (GraphPad Software, San Diego, CA, USA) were used for statistical analysis. Statistical significance was determined by the following *p*-values: P < 0.05; P < 0.01; P < 0.001.

## 3 Results

### 3.1 Non-diabetic mice: the influence of time, aging, and high sugar water intake on metabolic parameters

Using a longitudinal approach, glycemia, glycated albumin, and methylglyoxal levels were monitored from adulthood (T0, 9 months) to senescence (T4, 19 months), as illustrated in the experimental design (Figure 1). At T1, mice were repeatedly injected with either NaCl 0.9% (CTRL, n = 17) or STZ (50 mg/kg), STZ to induce diabetic conditions (DM, n = 18) (for details, see Materials and Methods, 2.3). Starting from T2, animals were randomized into two experimental groups: half of the mice were given with high-sugar water (CTRL-HS mice, n = 9; DM-HS mice, n = 9), while the remaining mice were continued with the normal diet of the animal facility (CTRL-ND mice, n = 8; DM-ND, n = 9). The two regimens were maintained for 5 months and monitored at chosen timepoints i.e., T3 and T4, 2 and 5 months after i.p. injections, respectively.

**FIGURE 2:**
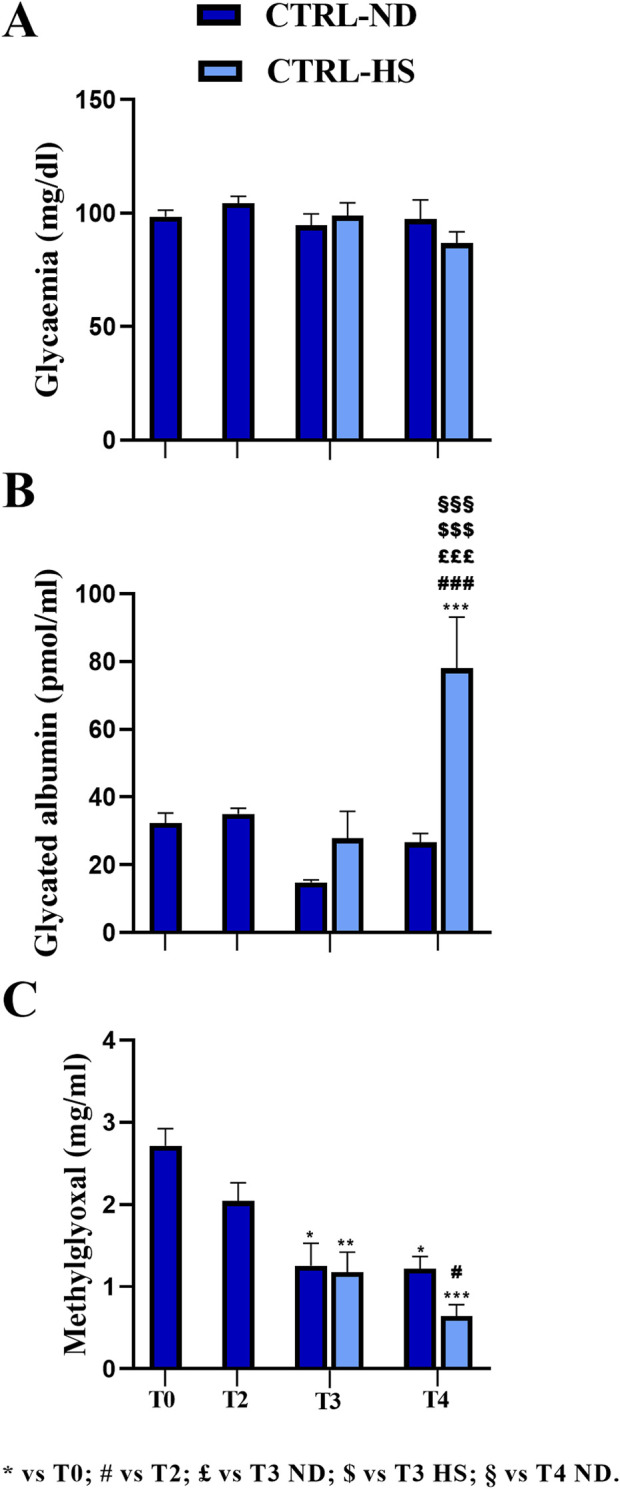
Comparison between normal (ND) and high sugar (HS) diet in control mice (CTRL) at different experimental time points. Fasting glycemia **(A)**, glycated albumin **(B)**, and methylglyoxal **(C)** levels were measured at T0 and T2 in CTRL mice and at T3 and T4 in CTRL-ND and CTRL-HS animals. Statistical significance (one-way ANOVA followed by Bonferroni *post hoc* test): * vs. T0, # vs. T2, £ vs. T3 ND, $ vs. T3 HS, § vs. T4 ND. For all symbols reported, p < 0.05 (*, #, £, $, and §); p < 0.01 (**, ##, ££, $$, §§); p < 0.001 (***, ###, £££, $$$, and §§§).

In CTRL mice, 1 month after i.p. injections of the physiological solution (T2), the mean glycemic value (104.45 ± 2.94 mg/dL, n = 8) was statistically comparable to that measured at T0 (98.44 ± 2.83 mg/dL, n = 35; Figure 2A). In addition, regarding GA, any difference recorded at T2 (35.05 ± 1.65 pmol/mL, n = 8) was comparable to that in T0 (29.13 ± 1.81 pmol/mL, n = 35; Figure 2B). Finally, the MGO mean value at T2 (2.04 ± 0.22 μg/mL, n = 8) was comparable to that calculated at T0 (2.72 ± 0.21 μg/mL, n = 35; Figure 2C).

At T3, the mean glycemic value of the CTRL-HS group (98.78 ± 5.76 mg/dL, n = 9) was comparable to that assessed in the CTRL-ND group (94.50 ± 5.15 mg/dL, n = 8). These data were further confirmed at T4, when the mean glycemic value of the CTRL-HS group (86.78 ± 5.06 mg/dL, n = 9) was comparable to that measured in the CTRL-ND group (97.29 ± 8.58 mg/dL, n = 8; Figure 2A). Hence, it follows that 5-month lasting (T4) HS supplementation did not elicit differences in fasting glycemic values, which were comparable to those determined in mice fed with a normal diet. Furthermore, from adulthood to senescence, any statistically significant difference in fasting glycemia was documented both in CTRL-ND and CTRL-HS groups.

Concerning the GA values, in CTRL-ND mice, any statistically significant difference was measured throughout the whole experimental duration, namely, from T0 to T4. At T3, the mean GA value in CTRL-HS animals (27.76 ± 8.0 pmol/mL, n = 9) was comparable to that calculated in CTRL-ND mice (14.77 ± 0.81 pmol/mL, n = 8). Notably, at T4, the mean GA value in CTRL-HS animals (78.08 ± 15.08 pmol/mL, n = 9) was statistically higher than that assessed in CTRL-ND mice at the same experimental timepoint (26.66 ± 2.62 pmol/mL, n = 8, *p*-value < 0.001; Figure 2B). Thus, the HS diet seemed to trigger a detrimental effect on GA values at T4 in the late senescence phase; notably, this mean value was statistically different from those measured at all the experimental timepoints in CTRL-ND mice (see Supplementary Table 1A).

Glycemic and GA mean values did not change during the lifespan, from adulthood to the senescence, as evidenced by comparing data gauged at all timepoints in CTRL mice fed with normal diet; differently, MGO significantly decreased during aging, as clearly detectable comparing mean values measured at T0 with those assessed at later timepoints (T0: 2.72 ± 0.21 μg/mL, n = 35; T2: 2.04 ± 0.22 μg/mL, n = 8; T3: 1.26 ± 0.27 μg/mL, n = 8, *p*-value = 0.017; T4: 1.22 ± 0.15 μg/mL, n = 8, *p*-value = 0.023; Figure 2C; Supplementary Table 1B). The same result was revealed in CTRL-HS mice (T3: 1.18 ± 0.24 μg/mL, n = 9, *p*-value = 0.005; T4: 0.64 ± 0.14 μg/mL, n = 9, *p*-value < 0.001, Figure 2C; Supplementary Table 1B). Interestingly, the mean MGO value measured in CTRL-ND mice was comparable to that determined in CTRL-HS animals.

Taken together, all the above reported data indicate that a 10% high sugar water intake alone did not exert detrimental effects on the three metabolic evaluated parameters until T3. Nonetheless, when prolonging intake of HS water for additional 3 months, the albumin glycation process increased significantly without any change in glycemic values, suggesting that the former is more sensitive to long-lasting HS intake. The decrease in MGO values during aging also persisted in animals belonging to the HS regimen group.

### 3.2 STZ-induced diabetes: the influence of time, aging, and high sugar water intake on metabolic parameters

**FIGURE 3:**
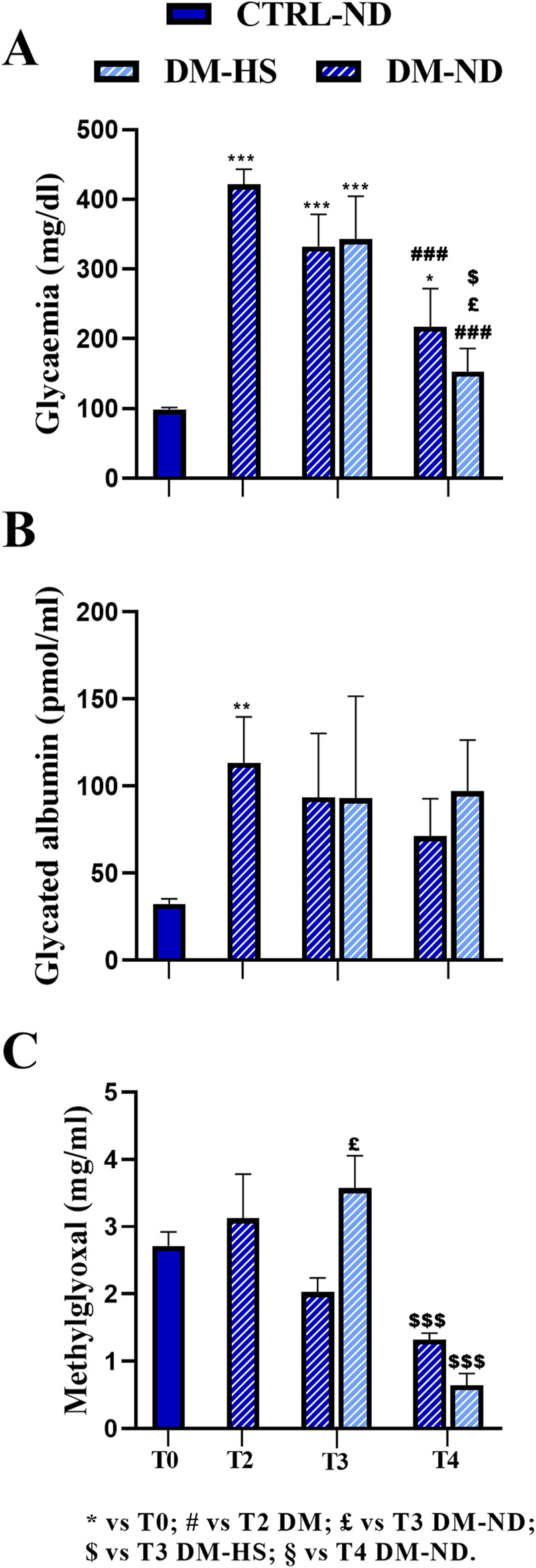
Comparison between normal (ND) and high sugar (HS) diet in control (CTRL) and diabetic mice (DM) at different experimental time points. Fasting glycemia **(A)**, glycated albumin **(B)**, and methylglyoxal **(C)** levels were measured at T2 in DM mice (n = 18) and at T3 and T4 in DM-ND and DM-HS animals. All data were compared to each other and compared to the value measured at T0 (n = 35). The value is reported as the mean ± standard error of the mean (SEM). Statistical significance: (one-way ANOVA followed by Bonferroni *post hoc* test): * vs. T0, # vs. T2 DM, £ vs. T3 DM-ND, $ vs. T3 DM-HS, § vs. T4 DM-ND. For all symbols reported, p < 0.05 (*, #, £, $, and §); p < 0.01 (**, ##, ££, $$, and §§); p < 0.001 (***, ###, £££, $$$, and §§§).

STZ induction was performed at T1 giving five consecutive i.p. injections during 5 subsequent days (DM-ND, n = 18). One month after STZ induction (T2), all STZ-induced mice displayed a dramatic statistically significant increase in the glycemic fasting values (on the mean, DM 421.5 ± 21.63 mg/dL, n = 18; Figure 3A) compared to T0 (98.44 ± 2.83 mg/dL, *p*-value < 0.001). It should be noted that all mice had a glycemic value higher than 300 mg/dL for three consecutive fasting measures in succeeding days, in accordance to literature data reporting a similar threshold value for the STZ-induced diabetic animal model (Graham et al., 2011; Liu et al., 2020; Furman, 2021). Hence, it was possible to classify these animals as diabetic mice (DM) in accordance with the Animal Models of Diabetic Complications Consortium (AMDCC). Notably, 1 month from STZ induction, the fasting glycemic mean value was definitely higher (412.7%) than that measured before the induction protocol.

Remarkably, at T2, the GA mean value was further dramatically increased (113.37 ± 26.39 pmol/mL, n = 18) compared to that assessed at T0 (29.13 ± 1.81 pmol/mL, n = 35, *p*-value = 0.0062; Figure 3B), even reaching a 389.6% increase in DM mice.

Diversely, the MGO mean value measured at T2 in the DM group (3.12 ± 0.66 μg/mL, n = 35) was comparable to that calculated at T0 (2.72 ± 0.21 μg/mL, n = 18).

Therefore, 1 month after STZ induction, both glycemia and GA increased compared to T0, while the mean MGO value remained relatively constant.

Notably, at T3, 2 months after the HS diet both DM-HS (342.78 ± 61.41 mg/dL, n = 9) and DM-ND mice (332.22 ± 46.40 mg/dL, n = 9; Figure 3A) showed a decrease in glycemic mean values, even though the value still remained statistically greater than that measured at T0 (*p*-value < 0.001; see Supplementary Table 2A). The glycemic mean value was further diminished at T4, after 5 months of HS diet, in both DM-HS (152.60 ± 33.25 mg/dL) and DM-ND (217.33 ± 54.54 mg/dL) mice, when compared with those assessed at T2 (Figure 3A; Supplementary Table 2A); any statistical significance was measured when comparing measures obtained at T4 with the mean value calculated at T0. The mean glycemic values assessed at T3 and T4 were not statistically different when comparing DM-ND and DN-HS mice. In conclusion, the effect of STZ induction on the glycemic value of DM mice was partially reversed 3 months after repeated injections, and the high sugar water intake had no detrimental effect on glycemic values at all checked experimental timepoints.

After an initial increase at T2, the GA mean value did not further change in a statistically significant manner at subsequent timepoints, namely, T3 and T4, in DM-ND (93.62 ± 36.61 pmol/mL, n = 9) and DM-HS mice (93.04 ± 58.61 pmol/mL, n = 9) and in DM-ND (71.22 ± 21.45 pmol/mL, n = 9) and DM-HS animals (97.16 ± 29.27 pmol/mL, n = 9), respectively (Figure 3B). In particular, the GA mean value still remained 3–4 times higher than that measured before STZ induction. Therefore, GA serves as a good biomarker for DM mice, mirroring the fasting glycemic value until T3. At T4, contrary to fasting glycemic values, GA did not decrease.

As in control mice, a tendency to decrease with time was revealed in DM animals, whose MGO mean values diminished from adulthood to senescence, without reaching statistical significance. At T3, a significant increase in MGO was measured in DM-HS mice (3.57 ± 0.48 μg/mL, n = 9) compared to DM-ND (2.03 ± 0.21 μg/mL, n = 9, *p*-value = 0.01). At T4, following an additional 3 months of high sugar water intake, the MGO mean value significantly decreased in DM-HS mice (0.64 ± 0.18 μg/mL) compared to that measured in the same animals at the previous timepoints (T3) (1.32 ± 0.10 μg/mL, *p*-value = 0.0009; Figure 3C; Supplementary Table 2B).

**FIGURE 4:**
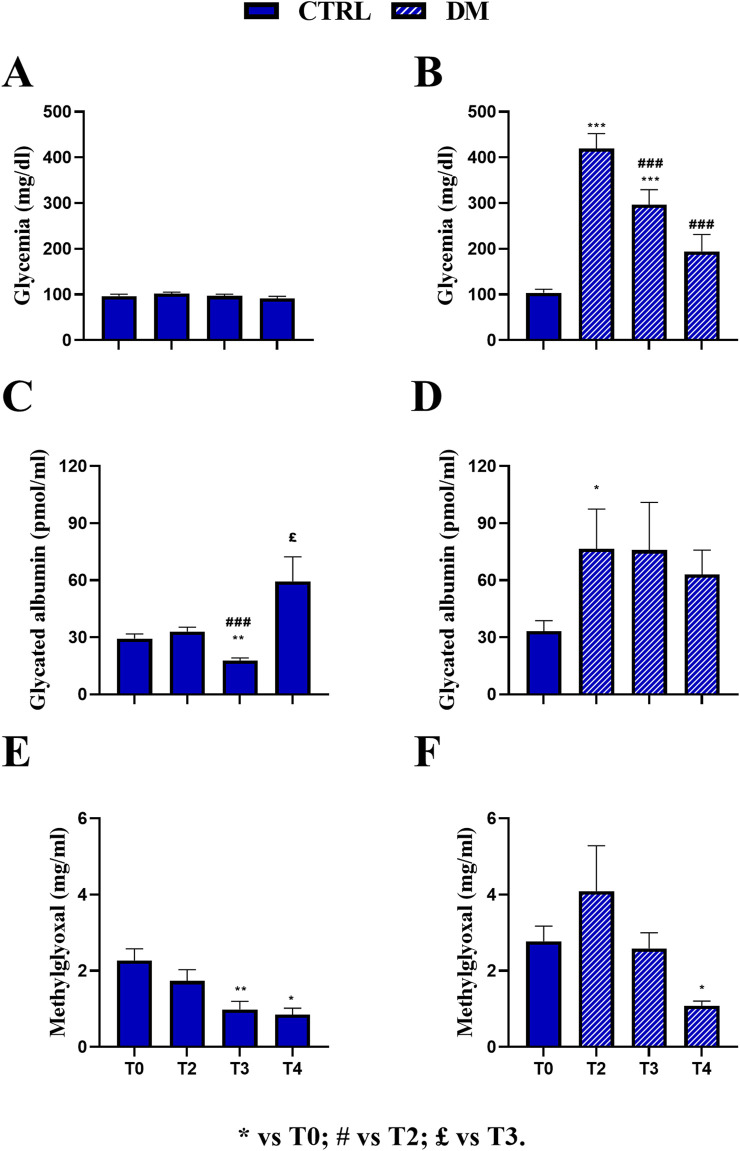
Comparison at all experimental times in control (CTRL) and diabetic (DM) mice. Fasting glycemia in CTRL **(A)** and DM **(B)**, glycated albumin in CTRL **(C)** and DM **(D)**, and methylglyoxal in CTRL **(E)** and DM **(F)** levels were assessed at T2, T3, and T4. The values are reported as the mean ± standard error of the mean (SEM). Statistical significance (repeated measures ANOVA followed by Bonferroni *post hoc* test): * vs. T0, # vs. T2, £ vs. T3. For all symbols reported, p < 0.05 (*, #, £); p < 0.01 (**, ##, and ££); p < 0.001 (***, ###, and £££).

As previously described, HS had no statistically significant effect on glycemic values both in CTRL and DM mice. Therefore, we pulled together the data regardless of the diet regimen at T3 and T4 to detect the differences within the two experimental groups CTRL and DM (Figure 4), analyzing the data by repeated measures ANOVA followed by the Bonferroni *post hoc* test from T0 to T4. For this reason, the dead animals at T4 were excluded from the analysis (CTRL: n = 16; DM: n = 14).

In the CTRL group, no significant variations were observed across T0 (97.5 ± 4.05 mg/dL, n = 16), T2 (103.31 ± 3.02 mg/dL, n = 16), T3 (97.75 ± 3.91 mg/dL, n = 16), and T4 (91.37 ± 4.73 mg/dL, n = 16), indicating stable fasting glycemia throughout the experimental period (Figure 4A). Conversely, the DM group exhibited a marked and statistically significant increase in glycemia at T2 (419.87 ± 32.38 mg/dL, n = 14) compared to T0 DM (103.64 ± 7.69 mg/dL, n = 14, *p-value* < 0.001; Figure 4B) and also compared to CTRL animals at the same timepoint (*p*-value < 0.001; Supplementary Table 3A; Supplementary Figure 2A). At T3, the mean glycemic value decreased in DM mice (296.57 ± 32.61 mg/dL, n = 14) compared to both T0 (*p-value* < 0.001) and T2 DM (*p*-value < 0.001), nonetheless differing from that assessed in CTRL mice at the same time point (*p*-value < 0.001; Supplementary Table 3A; Supplementary Figure 2A). Furthermore, at T4, the mean glycemic values in DM mice (194.21 ± 37.04 mg/dL, n = 14) further decreased compared to those at T2 (*p*-value < 0.001) and were also significantly different from those of CTRL mice at the same time point. In contrast, no statistically significant difference was assessed when compared to T0 (Supplementary Table 3A; Supplementary Figure 2A).

Regarding mean GA values, the CTRL group showed a significant increase at T4 (59.46 ± 12.89 pmol/mL) compared to T0 (29.37 ± 2.33 pmol/mL, *p-value* = 0.007). In contrast, no significant changes were detected at T2 (33.03 ± 2.36 pmol/mL), and a statistical decrease was observed at T3 (17.76 ± 1.35 pmol/mL) compared to both T0 and T2 (*p-value* < 0.001 and *p-value* = 0.03, respectively; Figure 4C). Remarkably, as described previously, the CTRL GA value was mainly affected by the HS regimen at T4. In the DM group, a significant increase in GA levels was observed at T2 (76.59 ± 20.87 pmol/mL) compared to T0 (33.29 ± 5.47 pmol/mL, *p-value* = 0.02) and CTRL mice at the same time point (*p*-value = 0.0064; Supplementary Table 3B; Supplementary Figure 2B). GA levels were subsequently decreased at T3 (75.82 ± 25.08 pmol/mL) and T4 (63.07 ± 12.76 pmol/mL) in DM mice, although statistical significance was not maintained across all time points (Figure 4D). However, a statistically significant difference was revealed between DM and CTRL mice at T3 (*p*-value = 0.043; Supplementary Table 3B; Supplementary Figure 2B).

Regarding the mean MGO value, the CTRL group showed a significant reduction at T3 (0.98 ± 0.21 μg/mL) and T4 (0.84 ± 0.17 μg/mL) compared to T0 (2.27 ± 0.30 μg/mL, *p-value* = 0.009 and *p-value* = 0.03, respectively), while no significant change was detected at T2 (1.73 ± 0.29 μg/mL; Figure 4E). Similarly, in the DM group, MGO levels significantly decreased at T4 (1.07 ± 0.12 μg/mL) compared to T0 (2.77 ± 0.40 μg/mL, *p-value* = 0.01; Figure 4F). Furthermore, no statistically significant increase was measured at T2 in DM mice (4.09 ± 1.18 μg/mL) compared to CTRL mice. However, at T3, a statistically significant increase was assessed in DM animals (2.58 ± 0.41 μg/mL) compared to CTRL mice (*p-value* = 0.0003; Supplementary Table 3C; Supplementary Figure 2C). At T4, the mean MGO value in DM mice was compared to that calculated in CTRL animals. It should be underlined that at T3, the mean MGO value was primarily affected by the HS regimen in DM mice, as previously reported.

### 3.3 Diabetic condition influenced weight, drinking, and survival probability

**FIGURE 5:**
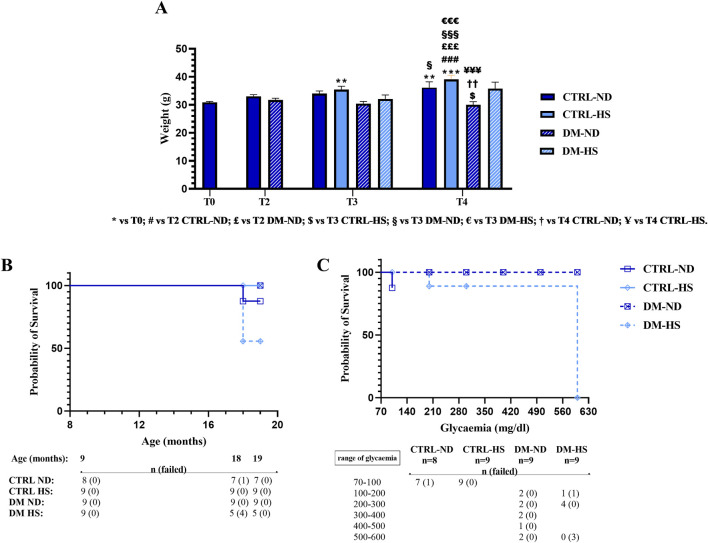
Weight monitoring and Kaplan–Meier survival analysis. Panel **(A)**: weight measured at T0 in controls (CTRL), at T2 in CTRL and diabetic (DM) mice, and at T3 in CTRL and DM both in normal (ND) and high sugar (HS) diet conditions. Values are presented as mean ± SEM. Panel **(B)**: Kaplan–Meier survival analysis showing survival curves of CTRL and DM mice, fed with either ND or HS diet. It should be noted that the DM-ND and CTRL-HS symbols are overlapped. In the lower part of panel B, the whole number of mice (n) and the number of dead mice (in brackets), at different ages, are reported. Panel **(C)**: Kaplan–Meier analysis presenting survival curves of DM mice, fed with either ND or HS diet, relative to the glycemic value assessed at 18 months of age (T3). In the lower part of panel C, the number of alive mice (n) and the number of dead mice (in brackets) are listed. Statistical significance for data presented in Panel A (two-way ANOVA followed by Bonferroni *post hoc* test): * vs. T0, # vs. T2 CTRL-ND, £ vs. T2 DM-ND, $ vs. T3 CTRL-HS, § vs. T3 DM-ND, € vs. T3 DM-HS, † vs. T4 CTRL-ND, and Ұ vs. T4 CTRL-HS. For all symbols reported, p < 0.05 (*, #, £, $, §, €, †, and Ұ); p < 0.01 (**, ##, ££, $$, §§, €€, ††, and Ұ Ұ); p < 0.001 (***, ###, £££, $$$, §§§, €€€, †††, and Ұ Ұ Ұ). Panel **(B, C)**: statistics for Kaplan–Meier analysis was obtained with a log-rank (Mantel–Cox) test.

The influence of diabetic conditions on body weight was investigated in all experimental groups at different timepoints (T0, T2, T3, and T4; Figure 5A).

At 9 months of age (T0), mice weighed 30.89 ± 0.33 g (n = 35). During aging, from adolescence to senescence, mice fed with the animal facility’s ND displayed an increase in weight gain (T2: 33.6 ± 0.58 g, n = 17; T3: 34 ± 0.93 g, n = 8; and T4: 36.14 ± 2.10 g), and this increase was gaged statistically significant at T4 compared to T0 (*p-value* < 0.001). On the contrary, DM mice fed with the same ND lost weight during aging (T2: 31.72 ± 0.61 g, n = 18; T3: 30.44 ± 0.80 g, n = 9), and this decrease became statistically significant at T4 (30.00 ± 1.13 g, n = 9) when compared to CTRL-ND mice evaluated at the same experimental time point (*p-value* = 0.0086).

During aging, both at T3 and T4, the weight of CTRL mice fed with HS tended to increase more than that measured in CTRL-ND mice, but any statistical significance was determined (CTRL-HS: 35.44 ± 1.19 g and 39.11 ± 1.46 g at T3 and T4, respectively) (Figure 5A; Supplementary Table 4). During aging, any statistically significant weight change was assessed in DM mice fed with HS (T3: 32.11 ± 1.40 g, n = 9; T4: 35.80 ± 2.31 g n = 5), suggesting that HS water intake counterbalanced the weight loss seen in DM-ND mice.

The water intake of diabetic mice (about 9 mL/day) increased by two-fold compared to that of control mice (approximately 4 mL/day). The weight loss and the higher water intake confirmed that this preclinical model had the same features of human diabetes (Liu et al., 2019).

The Kaplan–Meier survival analysis was performed in all experimental conditions during the mouse lifespan (Figure 7B) to monitor the survival probability (%). Comparing CTRL-ND, CTRL-HS, DM-ND, and DM-HS survival curves, a statistical significance was evidenced (*p-value* = 0.0241), indicating that both variables, i.e., HS water intake and diabetes induction, were crucial for survival probability.

It should be noted that the survival probability obtained by a direct comparison between CTRL-ND and DM-ND mice is similar, suggesting that diabetes alone was insufficient to influence the survival of animals up to the senescence phase (19 months of age). Furthermore, the survival probability obtained by a direct comparison between CTRL-ND and CTRL-HS mice indicated that HS alone did not alter the survival of control animals. Furthermore, a statistically significant difference between DM-ND and DM-HS mice was gaged (*p-value* = 0.0275), showing that HS affected the survival of diabetic animals. Additionally, a statistically significant difference was determined in survival rates while comparing CTRL-HS and DM-HS mice (*p-value* = 0.0275), indicating that diabetes modified the survival of animals treated with HS.

Therefore, we can conclude that the association between the diabetic condition and HS water intake affects animals’ survival, with DM-HS mice displaying a lower survival likelihood compared to all other experimental groups. Specifically, at 18 months of age, the survival probability in the DM-HS group was 55.55%.

We investigated more in-depth the relationship between the Kaplan–Meier survival probability considering the fasting glycemic level at T3 and the mice’s fate. Comparing DM-ND and DM-HS survival curves, a statistical significance was revealed, indicating that the fasting glycemic values influenced the survival of diabetic animals (*p-value* = 0.0295; Figure 5C).

In particular, only DM mice displayed a fasting glycemic value ranging between 500 and 600 mg/dL (n = 5). The survival probability measured for DM-HS mice (n = 0/3) was 0%, while settled on a percentage of 100% for DM-ND (n = 2/2), demonstrating the detrimental effect of HS in DM mice. Therefore, fasting glycemic values were not sufficient to illustrate the death of diabetic mice.

### 3.4 Reversal of STZ-induced diabetes

**FIGURE 6:**
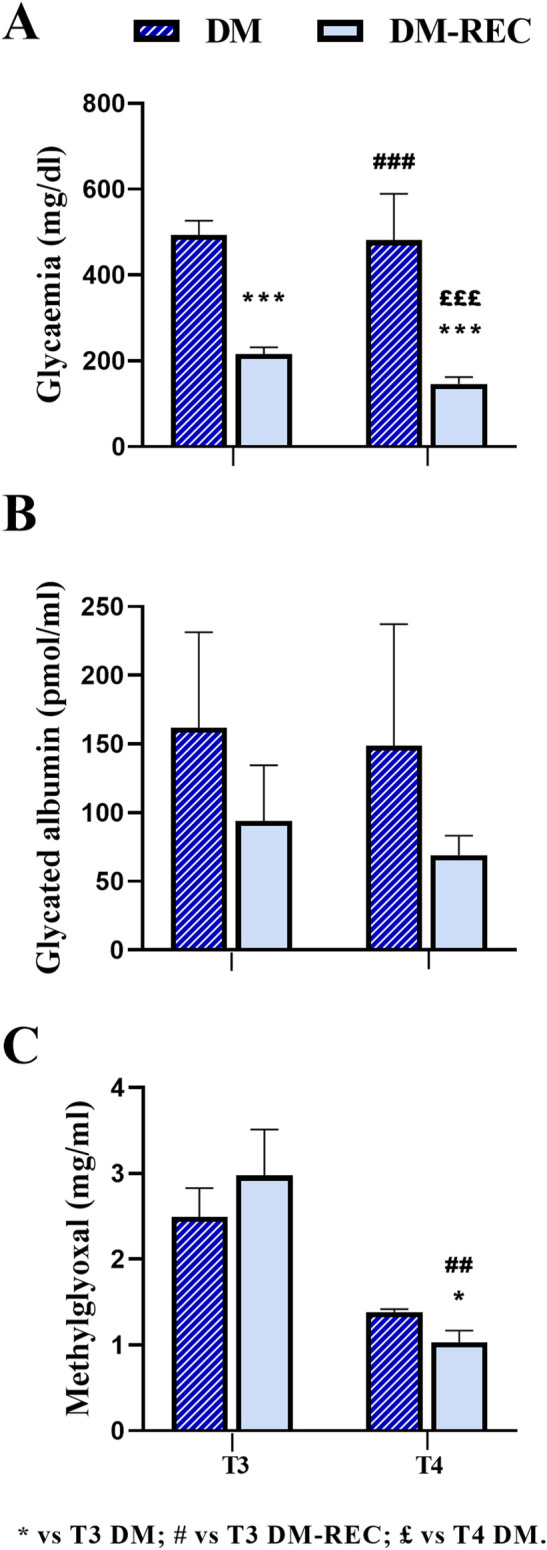
Comparison between diabetic mice (DM) and diabetic-recovery (DM-REC) animals at chosen experimental time points. Mice were divided into two subgroups: DM, with fasting glycemic values higher than 300 mg/dL, and DM-REC, with fasting glycemic values lower than 300 mg/dL: **(A)** fasting glycemic values, **(B)** fasting GA values, and **(C)** MGO values. Graphs showing the mean value line and the standard error of the mean (SEM) error bars. Statistical significance (unpaired T-test): * vs. T3 DM, # vs. T3 DM-REC, £ vs. T4 DM. For all symbols reported, p < 0.05 (*, #, and £); p < 0.01 (**, ##, and ££); p < 0.001 (***, ###, and £££).

Furthermore, with the aim of deepening the comprehension and clarifying the relationship between glycemia, GA, and MGO, the values assessed in DM mice (as the sum of DM-ND and DM-HS) are shown in Figure 6. The analysis revealed the scattering of data in both GA and MGO values.

According to the individual glycemic value and considering a threshold value of 300 mg/dL at T3, DM mice were divided into two groups: the DM-REC group (DM mice recovering normal glycemia) consisted of animals with glycemic values lower than the threshold level (mean value: 215.90 ± 15.82 mg/dL, n = 10, T3 DM RECA), whereas the DM group consisted of diabetic mice with a glycemic value greater than the threshold level (489.50 ± 35.81 mg/dL, n = 8, T3 DM; Figure 6). Interestingly, at T3, the glycemic values in DM mice were significantly different compared to those measured in DM-REC mice (*p-value* < 0.001).

Notably, at subsequent time points, namely, T4, only two mice maintained a glycemic value over the threshold (481 ± 108 mg/dL, n = 2, T4 DM). The remaining mice displayed a statistically significant decrease in glycemic values (146.42 ± 16.16 mg/dL, n = 12, T4 DM-REC, *p-value* = < 0.001; Figure 6A; Supplementary Table 5).

To verify if the glycemic value assessed at T3 was correlated to GA or MGO, the two above identified groups are plotted in Figures 6B, C. Any statistical difference was recorded for GA among the following: DM mice at T3 (161.79 ± 69.46 pmol/mL), DM-REC animals at T3 (93.96 ± 40.47 pmol/mL), DM mice at T4 (148.67 ± 88.54 pmol/mL), and DM-REC animals at T4 (69.12 ± 14.01 pmol/mL; Figure 6B). It should be noted that GA decreased at both T3 and T4 in DM-REC mice compared to DM mice; however, due to the scattered nature of the data, no statistical significance was observed.

On the contrary, MGO partially mirrored the glycemic effect, becoming significantly lower in DM-REC mice at T4 (1.03 ± 0.14 μg/mL) compared to those measured in DM animals at T3 (2.49 ± 0.34 μg/mL, *p-value* = 0.046) and DM-REC mice at T3 (2.98 ± 0.54 μg/mL, *p-value* = 0.002; Figure 6C).

### 3.5 Histological investigation of pancreatic islets revealed DM insulitis

Coronal pancreatic sections from CTRL, DM, and DM-REC mice were obtained at T4 and further processed for histological evaluation using H&E staining to estimate the potential DM-induced pancreatic alterations. A semiquantitative analysis has been conducted: a scoring system was utilized to evaluate the extent of tissue damage using conventional brightfield microscopy on a semiquantitative scale ranging from undetectable (0) to severe (4), according to Pavlovic et al. (2018).

In detail, the degree of lesions was recorded and graded as follows: score 0, normal islets; score 1, perivascular/periductal infiltration; score 2, peri-insulitis; score 3, mild insulitis (<25% of the islet infiltrated); score 4, severe insulitis (more than 25% of the islets infiltrated).

**FIGURE 7:**
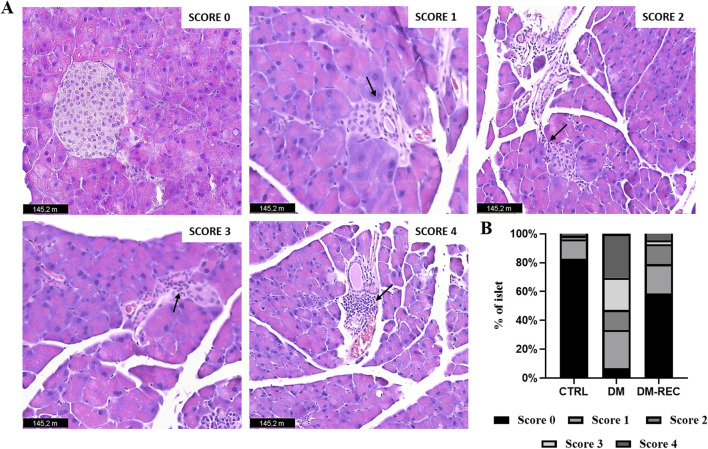
Recovering pathophysiology of pancreatic tissue in DM-REC mice after 6 months from STZ injections. Histological characterization via H&E staining. **(A)** Representative micrographs showing pancreas from different groups. Black arrows for each micrograph indicated the alterations observed for each score. Light microscopy magnification: ×40. Scale bars 145.2 m. **(B)** Insulitis was reduced in DM-REC mice compared to DM animals. Semiquantitative scale ranging from undetectable (0) to severe (4) tissue damage. In particular, degree of lesions was recorded and graded as follows: 0, normal islet; 1, perivascular/periductal infiltration; 2, peri-insulitis; 3, mild insulitis (<25% of the islet infiltrated); 4, severe insulitis (more than 25% of the islets infiltrated).

The examination showed a well-preserved physiological pancreatic cytoarchitecture in CTRL mice characterized by a high percentage (82.82%) of normal islets (score 0); differently, a low percentage (6.94%) of normal islets were assessed in DM mice. Notably, a partial recovery of regular islets (58.53%; Figures 7A, B) was recorded in DM-REC animals.

The occurrence of mild and severe insulitis was revealed in DM mice (22.22% and 30.56% for scores 3 and 4, respectively), while the infiltration decrease led to a percentage decrease in DM-REC animals (2.68% and 4.52% for scores 3 and 4, respectively). Finally, perivascular/periductal infiltration was documented in CTRL mice (13.37%) but enhanced in DM mice (26.96%) before decreasing again in DM-REC animals (20.41%).

Nevertheless, a comparable percentage of peri-insulitis (score 2) was determined in DM mice and DM-REC animals (13.89% and 13.87% for DM and DM-REC, respectively), whereas the percentage was definitely lower in CTRL mice (2.51%). Therefore, in DM mice, the harsh and time-persistent effects of STZ accounted for a high percentage of high-score insulitis in pancreatic sections, which was partially recovered in the histological profile assessed in DM-REC mice (Figures 7A, B).

### 3.6 Recognition memory and metabolic parameters: is there any relationship?

To study the possible relationship between changes in metabolic parameters, diabetes induction, and recognition memory’s “knowledge component,” specific behavioral tests were performed at different experimental time points (see *Materials and Methods*). To match the chosen metabolic parameters with recognition performance parameters, the above reported longitudinal approach and experimental time points (i.e., T0, T3, and T4) were maintained.

Concerning behavioral tests in CTRL and DM mice, similarly to the trend obtained for metabolic parameters, any difference was assessed neither in ND nor in HS diet condition (data not shown). Hence, collecting data together regardless of the diet regimen, the two experimental groups, namely, CTRL and DM were compared. Furthermore, considering DM mice and the previously reported difference in fasting glycemic values measured at T3, mice were divided into DM (fasting glycemic value higher than 300 mg/dL) and DM-REC groups (fasting glycemic value lower than 300 mg/dL) (Figure 6A).

**FIGURE 8:**
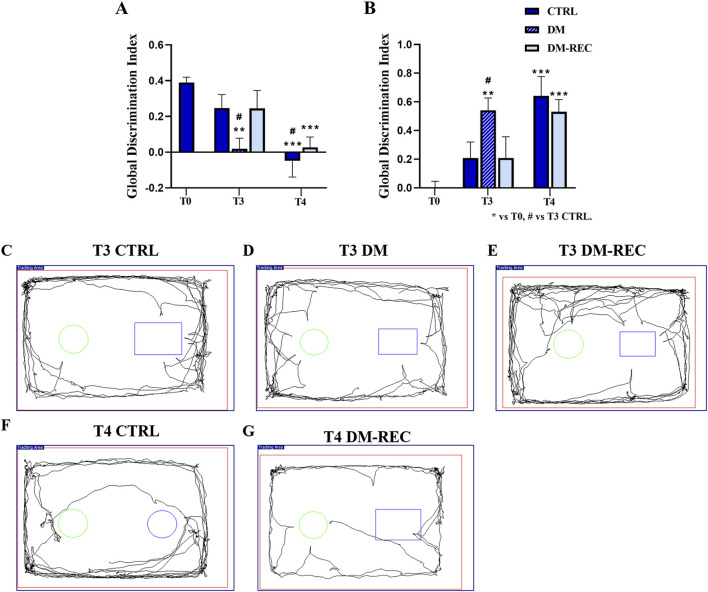
Diabetes exacerbates the cognitive decline monitored during aging: novel object recognition test (NOR) data. Panel **(A, B)**: global discrimination index (DI) and global frailty index (FI), respectively. Panel **(C–E)**: representative maps in CTRL **(C)**, DM **(D)**, and DM-REC **(E)** mice obtained at T3. Panel **(F)** and **(G)**: representative maps in CTRL **(F)** and DM-REC **(G)** mice acquired at T4. Values are presented as the mean ± standard error of the mean (SEM). Statistical significance (one-way ANOVA followed by Bonferroni *post hoc* test): * vs. T0, # vs. T3 CTRL, £ vs. T3 DM, $ vs. T3 DM-REC, § vs. T4 CTRL. For all symbols reported p < 0.05 (*, #, £, $, and §); p < 0.01 (**, ##, ££, $$, and §§); p < 0.001 (***, ###, £££, $$$, and §§§).

The discrimination index (DI) of the parameters obtained from the NOR task (see *Materials and Methods*) was analyzed, and subsequently, a global DI was generated (Figure 8A) together with the corresponding global frailty index (FI) (Figure 8B). Furthermore, Figure 8 presents examples of movement tracking for a single mouse at T3 in CTRL (Figure 8C), DM (Figure 8D), and DM-REC (Figure 8E) groups, and at T4 in CTRL (Figure 8F) and DM-REC (Figure 8G) groups.

During aging, from adulthood (T0) to senescence (T4), the expected physiological decline in recognition memory was recorded in CTRL mice (Ratto et al., 2019), in which the global FI changed from 0.00 ± 0.04 at T0 (n = 35), 0.21 ± 0.11 at T3 (n = 12), and 0.64 ± 0.13 at T4 (n = 12) (Figure 8B).

After 3 months (T3), STZ induction caused a decline in the recognition memory performance of DM mice, influencing both discrimination and the frailty index (Figures 8A, B). In particular, the global frailty index in DM mice at T3 (0.54 ± 0.09, n = 8) was significantly different compared to that measured in CTRL mice at the same time point (*p-value* = 0.04) (Figure 8B; Supplementary Table 6). Concerning DM-REC mice at T3, a recovered global FI (0.21 ± 0.15, n = 10) was determined, which is comparable to that measured in CTRL mice at the same time point.

At T4, most STZ-induced mice exhibited recovery in glycemic (DM-REC mice) values, which was paralleled by recognition memory performance similar to that recorded in CTRL mice at the same time points (Figure 8B). Moreover, the detrimental effect of STZ induction was partially reverted as deductible by examining both the discrimination and global frailty index of the NOR test (0.53 ± 0.09, n = 12) (Figures 8A, B; Supplementary Table 6). The remaining DM mice (n = 3) displayed extremely low locomotor activity, approaching novel and familiar objects for 1–2 times in 5 min for less than 3 s each time. Therefore, the decrease in locomotor activity in DM mice at T4 prevented calculation of the frailty index.

### 3.7 Histological investigation of the coronal hippocampus section

**FIGURE 9:**
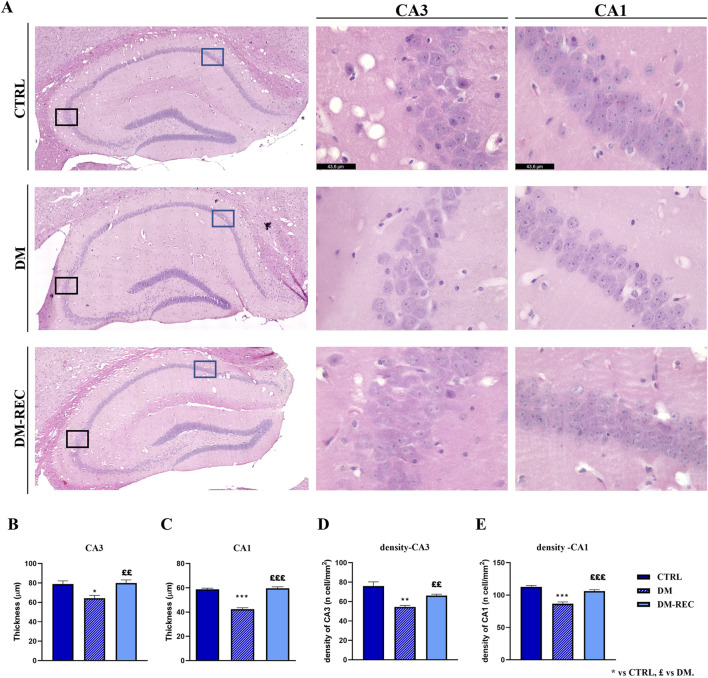
Histological characterization of hippocampus via H&E staining. Panel **(A)**: representative brain sections showing the hippocampal cytoarchitecture. *Left column*: the whole hippocampus images were obtained using the LASX Navigator. Black and blue squares indicate the ROI region for CA3 and CA1, respectively. *Center and right column*: high-magnification micrographs of CA3 and CA1, respectively, from CTRL, DM, and DM-REC mice. Light microscopy magnification: ×40. Scale bars 43.6 µm. Panel **(B–E)**: histograms showing the thickness of CA3 **(B)** and CA1 **(C)** and the cell density measured in CA3 **(D)** and CA1 **(E)** in CTRL, DM, and DM-REC mice. Values are presented as the mean ± standard error of the mean (SEM). Statistical significance (one-way ANOVA followed by Bonferroni *post hoc* test): * vs. CTRL, £ vs. DM. For all symbols reported p < 0.05 (*, £); p < 0.01 (** and ££); p < 0.001 (*** and £££).

Coronal brain sections from CTRL, DM, and DM-REC mice were obtained at T4 and further processed for histological evaluation using H&E staining to estimate the potential alterations in the hippocampus, being crucially involved in recognition and spatial memory, caused by STZ induction. The examinations focused on the dentate gyrus (DG) and the Ammon’s horn region, including CA1–CA3 subdivisions. Representative H&E micrographs are illustrated in Figure 9A.

The physiological gross morphology of the whole hippocampus was preserved in both experimental groups. The Ammon’s horn region was typically divided into four areas, namely, CA1, CA2, CA3, and CA4, the last of which was included in the V-shaped DG region. High-magnification images of DG from CTRL, DM, and DM-REC mice showed typical three well-defined layers, namely, molecular layer (ML), granule cell layer (GL), and pleomorphic layer (PL). Regarding the CA region, the characteristic three-layer organization was detected, consisting of the outer polymorphic layer (stratum oriens, SO), the middle pyramidal cell layer (stratum pyramidale, SP), and the inner molecular layer (stratum radiatum, SR).

Notably, the quantitative investigation evidenced area-specific alterations, evaluated in terms of both layer thickness and cell density, mainly localized in the CA1 and CA3 regions (Figure 9), while DG and CA2 remained unaffected (data not shown).

In detail, a significant decrease in the thickness of both CA1 (*p-value* < 0.001) and CA3 (*p*-value = 0.0113) of DM mice was revealed compared to CTRL animals. Simultaneously, a significant increase was measured in the thickness of both CA1 (*p-value* < 0.001) and CA3 (*p-value* = 0.0053) in DM-REC mice compared to DM mice (Figures 9B, C).

The calculation of cell density in the CA1 and CA3 regions evidenced a significant cell loss in DM mice compared to CTRL mice in both areas (*p-value* < 0.001 and 0.0011 for CA1 and CA3, respectively). Differently, the cell density increased in DM-REC animals compared to DM mice, both in CA1 (*p-value* = 0.0003) and in CA3 (*p-value* = 0.007) regions (Figure 9D).

## 4 Discussion

The present experimental work addresses the following five main key-points on STZ-induced diabetic mice model:

1. Monitoring fasting glycemia levels over time.
2. Investigating the efficacy of new blood biomarkers, such as fasting GA and MGO, in monitoring diabetic conditions and their relationship with fasting glycemia.
3. Exploring the potential detrimental effect of a high sugar water intake on diabetes conditions.
4. Considering the relationship between diabetic conditions and declarative, episodic long-term memory.
5. Examining histological features/alterations of both pancreas and hippocampus in diabetic mice.

A mouse model of type 2 diabetes was reproduced using the low-dose streptozotocin (STZ) induction protocol according to the Animal Models of Diabetic Complications Consortium (AMDCC) (Bolzán and Bianchi, 2002; Burdo et al., 2009). STZ is an antibiotic that causes partial or complete death of pancreatic β-cells and is widely used to induce diabetes in animal models. The high-dose STZ protocol induces a complete destruction of pancreatic β-cells, reproducing T1DM. On the contrary, low-dose STZ administration induces a gradual death of pancreatic β-cells, mimicking the pathogenesis of DM (Cassano et al., 2020; Furman, 2021; Lai et al., 2024). In the literature, several experimental protocols are described, which combine different STZ doses, number of intraperitoneal injections, and dietary regimens (Wu and Huan, 2008; Ventura-Sobrevilla et al., 2011; Cassano et al., 2020; Furman, 2021; Liu et al., 2021; Chen et al., 2022; Bauer et al., 2023b; Lai et al., 2024).

In this animal model, diabetes was induced using low doses of STZ at 13 months of age, corresponding to the late adulthood phase (T1), approximately equivalent to 43 human years (Dutta and Sengupta, 2016). One month later, at 14 months of age (T2) and still in the adulthood phase, comparable to 46 human years, all mice fed with ND (DM-ND) exhibited diabetic fasting glycemic levels (four times higher compared to controls). At T3, corresponding to the reproductive senescence phase (16 months of age in mice and equivalent to approximately 53 human years), 55.55% of the mice (n = 10/18) in both experimental groups DM-ND and DM-HS showed partial recovery, achieving fasting glycemic levels below the diabetic threshold (lower than 300 mg/dL). By T4, during the senescence phase (19 months of age in mice and roughly equivalent to 62 human years), 88.9% of the mice in DM-ND and DM-HS groups (n = 16/18) had recovered fasting glycemic levels below the diabetic threshold, which was statistically comparable to the results assessed before induction. These findings indicate that the diabetic mouse model remains stable in fasting glycemic values for 1 month, corresponding to three human years (from 43 to 46 years). During this timeframe, our results show a clear correlation with human data. However, as highlighted in the study, over the subsequent 2 months (equivalent to 7 human years, from 46 to 53 years), approximately 55% of the mice exhibited partial recovery of fasting glycemic levels, resulting in a reduced correlation with human data. Beyond this point, the correlation with human data was completely lost. In conclusion, this longitudinal study demonstrates that the diabetic mouse model maintains stability for a specific period but later exhibits biological variability, with some mice recovering glycemia by “itself.” This unexpected result could pave the way for further investigation into the cellular and molecular mechanisms underlying glycemic recovery, potentially uncovering novel therapeutic strategies for diabetes.

It should be noted that, as measured at T3 and T4, the HS diet did not exert any detrimental effect on the fasting glycemic levels, according to data on previous studies on adult mice (Coirini et al., 2022).

The STZ-induced diabetic mouse model has, therefore, a disease reversible over time, as demonstrated by the diabetic condition which is maintained in a short time window of only 3 months.

In parallel with the glycemia, we monitored the blood levels of GA and MGO. GA is the higher glycated portion of fructosamine and reflects short-term glycemic changes, which occur over a 3-week period (Freitas et al., 2017; Piuri et al., 2020). GA is evaluated in particular clinical conditions such as pregnancy or hemoglobinopathy or chronic kidney disease. In the last decades, based on the easy and fast standardized enzymatic methodology, GA has been suggested as a marker of DM screening and monitoring, as well as a predictor of long-term diabetic outcomes (Freitas et al., 2017). Our data indicate that, despite glycemic values in control animals remaining stable over time regardless of the dietary regimen, GA was statistically higher after 5 months of HS, suggesting that glycated albumin was affected by a long-term effect of HS water intake. In diabetic mice, GA was statistically increased 1 month after STZ i.p. injections and remained stable over time until T4, even though glycemic level decreased and recovered to a normal fasting value in most mice. Hence, GA seems to be a good blood biomarker with the same early onset of glycemia and a long-lasting response compared to fasting glycemia. GA measured in STZ-induced mice was similar in both DM-HS and DM-ND animals, suggesting that diabetes induction had a greater impact on HS water intake. These data suggest that even as glycemia normalized, ongoing metabolic disturbances persisted. GA serves as an important biomarker of short-term glycemic control, and its elevation indicates that chronic hyperglycemic conditions lead to prolonged protein glycation processes.

MGO is a highly reactive dicarbonyl compound, mainly formed as a byproduct of glucose metabolism. MGO is the major precursor of nonenzymatic glycation of proteins and DNA, subsequently leading to the formation of advanced glycation end products (AGEs). MGO is an emerging biomarker of DM due to its strong association with protein glycation and insulin resistance (Ramachandra Bhat et al., 2019). Therefore, monitoring plasma MGO levels could be useful for investigating disease-related complications in diabetic patients. In particular, high MGO levels correlate with chronic kidney disease, macroangiopathy, and cognitive deficits (Ogawa et al., 2010; A; Shamsaldeen et al., 2016).

Surprisingly, in control mice, fasting MGO serum levels tended to decrease during aging in a statistically significant manner, regardless of the dietary regimen followed. Under physiological circumstances, MGO is detoxified by the glyoxalase system consisting of glyoxalase I and II (Glo1 and Glo2). We can speculate that the serum MGO level is inversely correlated to the intracellular MGO level. Few studies have been conducted to explore the effects of aging on MGO serum levels in mice. MGO is highly reactive and can bond with endogenous nucleophilic substances and can be eliminated by the glyoxalase system or form stable AGEs in the cell. In particular conditions, when the glyoxalase system function is compromised, intracellular MGO levels increase and produce intracellular AGEs, leaving little MGO in plasma or serum (Dhar and Desai, 2012; Kold-Christensen and Johannsen, 2020). Furthermore, some investigations reported that patients with type 1 and type 2 diabetes have higher MGO plasma levels compared to healthy individuals (McLellan et al., 1994; Wang et al., 2007). Interestingly, MGO serum levels increased in DM-HS evaluated at T3, correlating the described decrease with aging in CTRL mice. At T4, when glycemic levels recovered to the normal value, MGO decreased, mirroring the fasting glycemic trend.

Concerning histological evaluation, the examination of pancreatic tissue sections confirmed that the STZ-induced diabetic condition was characterized by pathological infiltration leading to insulitis. Interestingly, in DM-REC mice, the recovery in glycemia was mirrored by a partial recovery of normal histological features. This partial recovery could indicate ongoing β-cell regeneration mechanisms, which may include β-cell replication, neogenesis, and inhibition of apoptosis (Trucco, 2005; Levine and Itkin-Ansari, 2008). Studies suggest that the recovery could be caused by different mechanisms, including proliferation via pathways like IRS2/PI3K/Akt (Ji et al., 2022), neogenesis from precursor cells (Shahedi et al., 2024), and trans-differentiation of α-cells into β-cells (Dor et al., 2004). Additionally, an antiapoptotic mechanism and immunomodulation, possibly involving the spleen, could play an indirect role in promoting regeneration (Yin et al., 2006). Our murine model of diabetes appears to be particularly useful for studying and further exploring the mechanisms involved in the recovery from the diabetic condition.

The combination of diabetes and HS has a greater impact on decreasing the survival probability. In fact, the survival probability measured in 18-month-old DM-HS mice was 55.55%. In particular, the survival probability of mice with fasting glycemic value in the range 500–600 mg/dL was 0%. Otherwise, DM-ND mice with a high glycemic value survived, indicating that diabetic animals on the HS dietary regimen have a lower survival likelihood compared to other experimental groups evaluated in the study.

In the novel object recognition task, the recognition memory performance was dramatically impaired in DM mice at T3, underlying the strong relationship between diabetes and cognition. In particular, the global FI at T3 in DM mice was more than doubled compared to that calculated in control animals at the same age, reaching a value comparable to that estimated at T4, which reflects cognitive decline similar to that observed in late senescent non-diabetic mice. Interestingly, at T3, DM-REC mice recovered the memory task performance, obtaining a global FI comparable to that measured in CTRL mice at the same time point. This finding suggests that the STZ-induced decline in recognition memory could be reverted if glycemia is reverted, possibly through enhanced neuroprotective mechanisms or neurogenesis. At T4, DM mice became phenotypically frail with a dramatic decline in the locomotor activity. The hippocampus is a crucial anatomical structure that plays a significant role in learning, memory, and various cognitive functions. Hence, the study of this brain area is crucial for assessing cognitive dysfunction associated with diabetes (Hu et al., 2019; Li et al., 2020).

Based on histological examination, our data in DM mice evidenced STZ-induced cytotoxic injuries, mainly detectable in the hippocampal CA1 and CA3 regions, as demonstrated by both thickness reduction and cell density decrease. Interestingly, the detrimental effect of STZ was reverted in the DM-REC mice, with an increase in both thickness and cell density, in line with the recovery phenomena measured by fasting glycemia and recognition memory performance. Thus, these findings further demonstrated the neural plasticity of these brain areas which regained a physiological feature.

The glycemic level following low-dose STZ induction, whether associated with normal diet regimen or high-sugar intake, reversed over time in 5 months, allowing us to explore the cellular mechanism involved/underlying the recovery phenomena observed both in the pancreas and hippocampus. Potential mechanisms may include enhanced β-cell regeneration and neurogenesis in the pancreas, paralleled by a recovery in hippocampus memory neuronal networks. All these mechanisms require further investigation.

## 5 Conclusion

In conclusion, the currently described STZ induction protocol induces a non-lasting diabetic condition, as evidenced by fasting glycemia and glycated albumin increase. GA should be considered a novel and emerging biomarker for diabetic conditions in mice, with a similar early onset of hyperglycemia but with a longer duration. The survival probability is inversely related to both HS and diabetic conditions. The two hallmarks of the diabetic condition are the presence of pancreatic tissue insulitis and the specific alterations in CA1 and CA3 hippocampal regions. The impairment in recognition memory mirrored hyperglycemia in diabetic mice. The cytoarchitecture of the pancreas and hippocampus and recognition memory showed signs of recovery.

Interestingly, an improvement in recognition memory and a partial regaining of physiological pancreas morphology and hippocampal cytoarchitecture are recorded in mice with recovered fasting glycemia levels,

## Data Availability

The original contributions presented in the study are included in the article/Supplementary Material; further inquiries can be directed to the corresponding author.
