# Supplementary material for: A pathophysiological intersection between metabolic biomarkers and memory: a longitudinal study in the STZ-induced diabetic mouse model
Source: Front Physiol. 2025 Mar 12;16:1455434. doi: 10.3389/fphys.2025.1455434 (PMC11937145; doi:10.3389/fphys.2025.1455434)
Supplement: Supplementary file 1 [file DataSheet1.docx]

Supplementary Material

A pathophysiological intersection between metabolic biomarkers and memory: a longitudinal study in STZ- induced diabetic mouse model.

Maria Teresa Venuti, Elisa Roda, Federico Brandalise, Meghma Sarkar, Mattia Cappelletti, Attilio F. Speciani, Irene Soffientini, Erica Cecilia Priori, Francesca Giammello, Daniela Ratto, Carlo Locatelli, and Paola Rossi^*^

*** Correspondence:** Paola Rossi: [paola.rossi@unipv.it](mailto:paola.rossi@unipv.it)

# Supplementary Tables

**
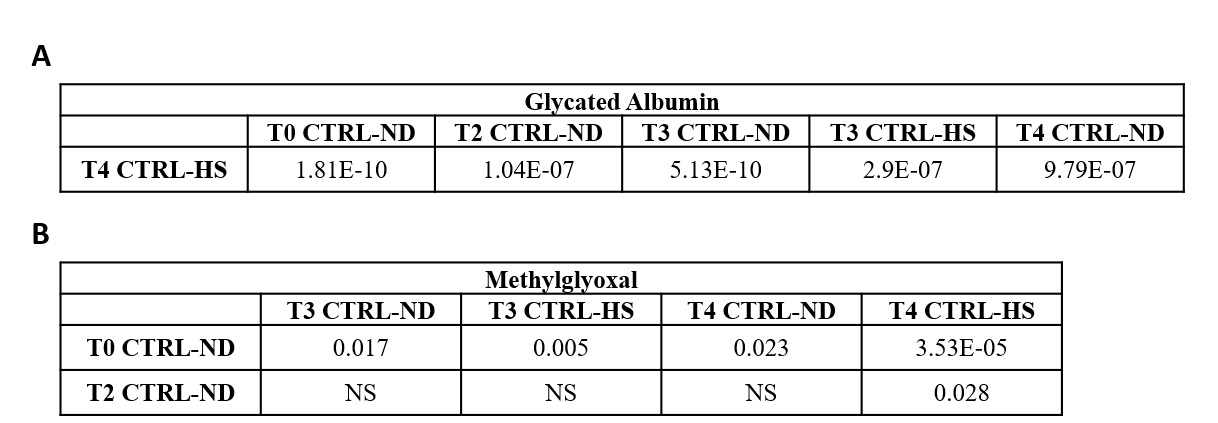
**

**Supplementary Table 1. Comparison between normal (ND) and high sugar (HS) in control mice (CTRL) at different experimental times***. p*-value for Glycaemia and Methylglyoxal obtained using One-Way ANOVA followed by Bonferroni *post-hoc* test**.**

**
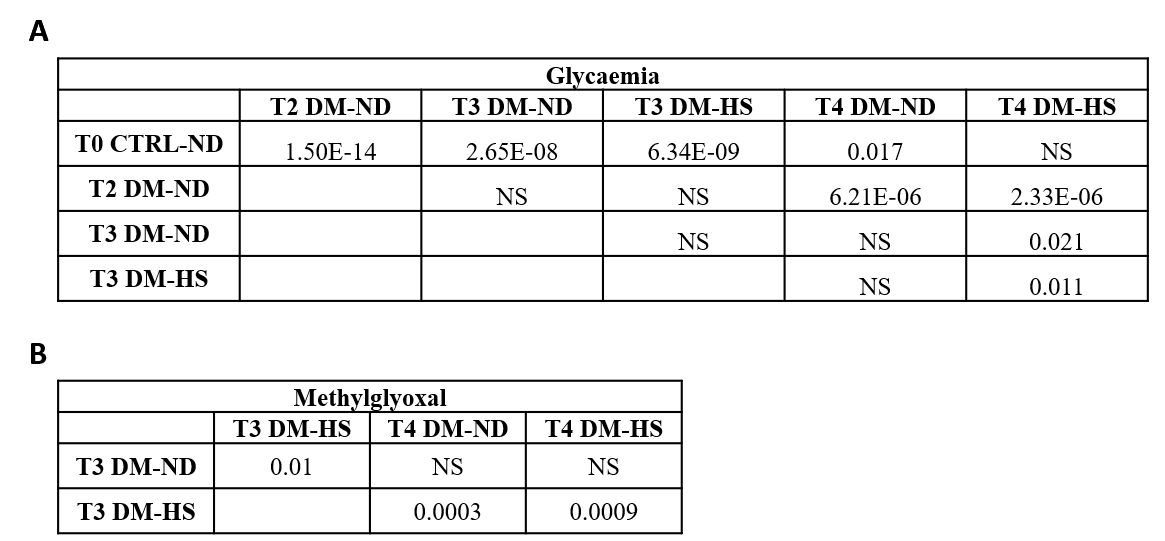
**

**Supplementary Table 2. Comparison between normal (ND) and high sugar (HS) in diabetic mice (DM) at different experimental times***. p*-value for Glycaemia and Methylglyoxal obtained using One-Way ANOVA followed by Bonferroni *post-hoc* test**.**

**
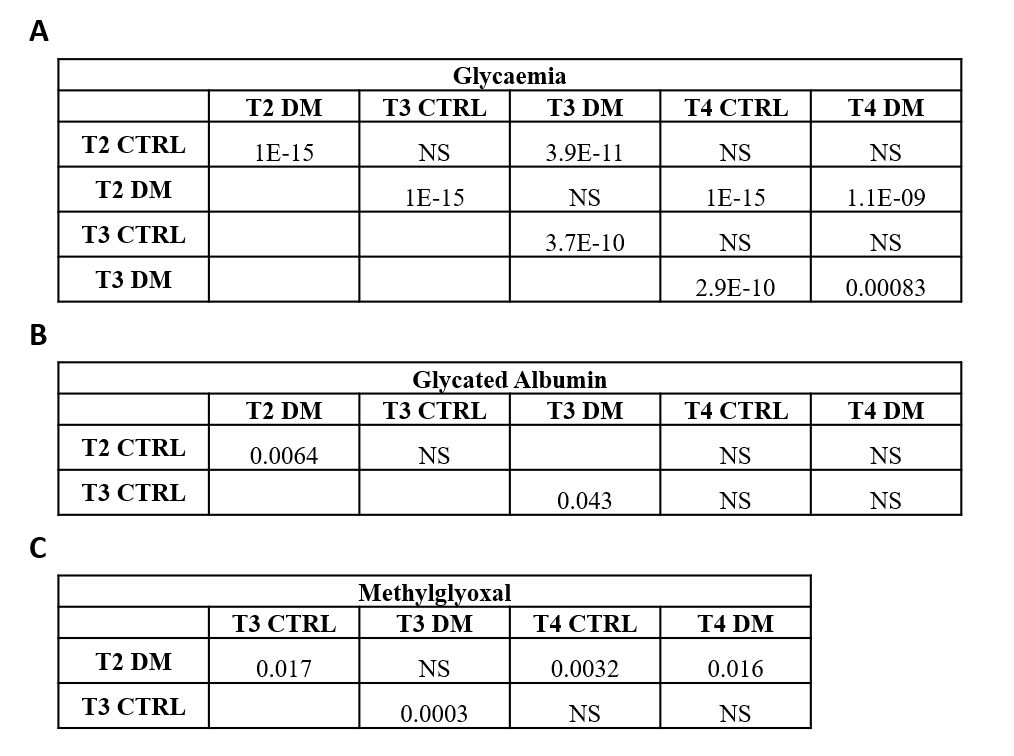
**

**Supplementary Table 3. Comparison between control (CTRL) and diabetic mice (DM) at different experimental times.**  *p*-value for Glycaemia, Glycated albumin, and Methylglyoxal obtained using One-Way ANOVA followed by Bonferroni *post-hoc* test**.**

**
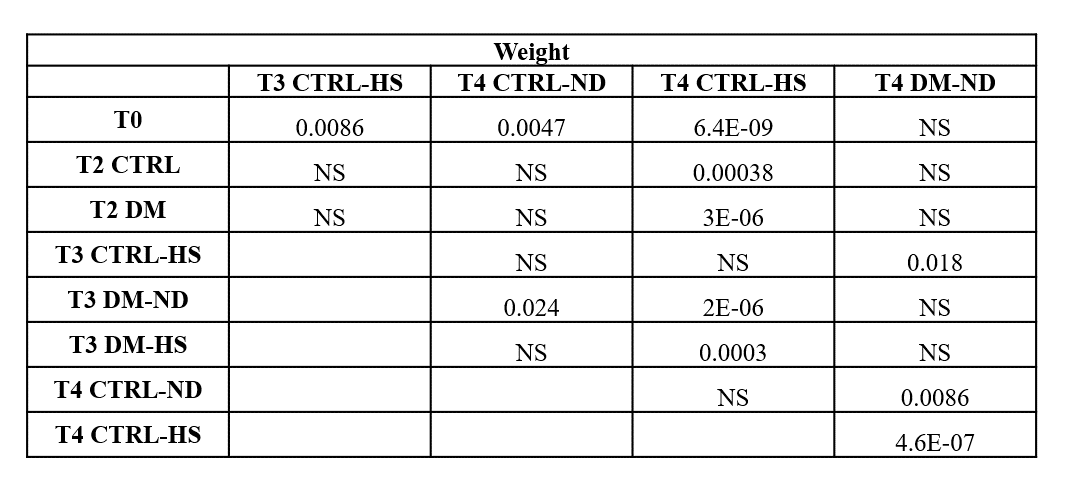
**

**Supplementary Table 4. Weight monitoring of and Kaplan-Meier survival analysis.** *p*-value for Weight obtained using One-Way ANOVA followed by Bonferroni *post-hoc* test**.**

**
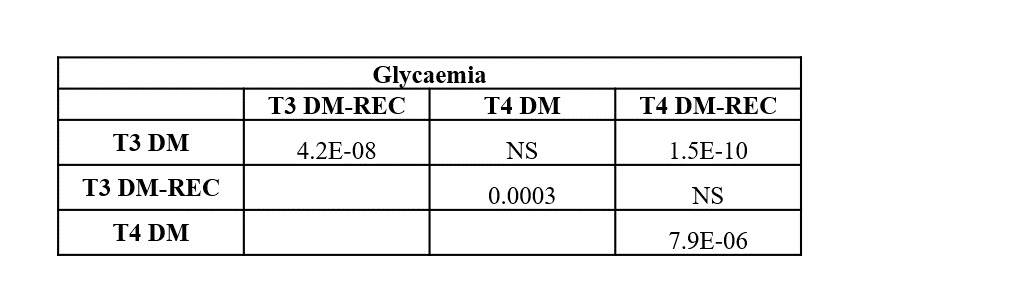
**

**Supplementary Table 5. Comparison between diabetic mice (DM) and diabetic-recovery (DM-REC) animals at chosen experimental timepoints.** *p*-value for Glycaemia obtained using One-Way ANOVA followed by Bonferroni *post-hoc* test**.**

**
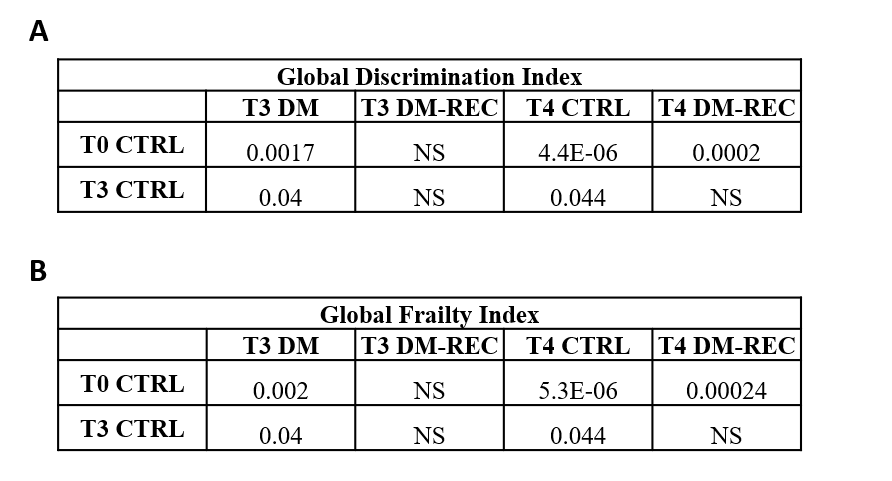
**

**Supplementary Table 6. Diabetes exacerbates the cognitive decline monitored during aging: Novel Object Recognition test (NOR) data.** *p*-value for Global Discrimination Index and Global Frailty Index obtained using One-Way ANOVA followed by Bonferroni *post-hoc* test**.**

# Supplementary Data

The OGTT test was performed at T0 in CTRL mice (n=35), and at T2 in CTRL (n=17) and DM mice (n=18), one month after STZ induction, as shown in figure 1 below. The mice were fasted overnight and then administered an oral gavage of glucose solution (Glucosio Sclavo, diagnostics 75 g/150 ml) at a dosage of 1 g/kg of body weight (Ayala et al., 2010; Nagy and Einwallner, 2018). The blood glucose level was measured at 0 (fasting glycemia) and every 30 minutes after ingestion until 3 hours. Using ORIGIN 6.0 software, the response glucose curve and the area under the curve (AUC) were calculated for each mouse tested, and then the mean and SEM of the curve and AUC were determined. Specifically, the OGTT was statistically different in DM mice at T2 compared to CTRL mice at T0 (p-value = 6.92 x 10-13) and T2 (p-value = 1.26 x 10-11), as shown by the area under the glycemic curve (AUC, B).


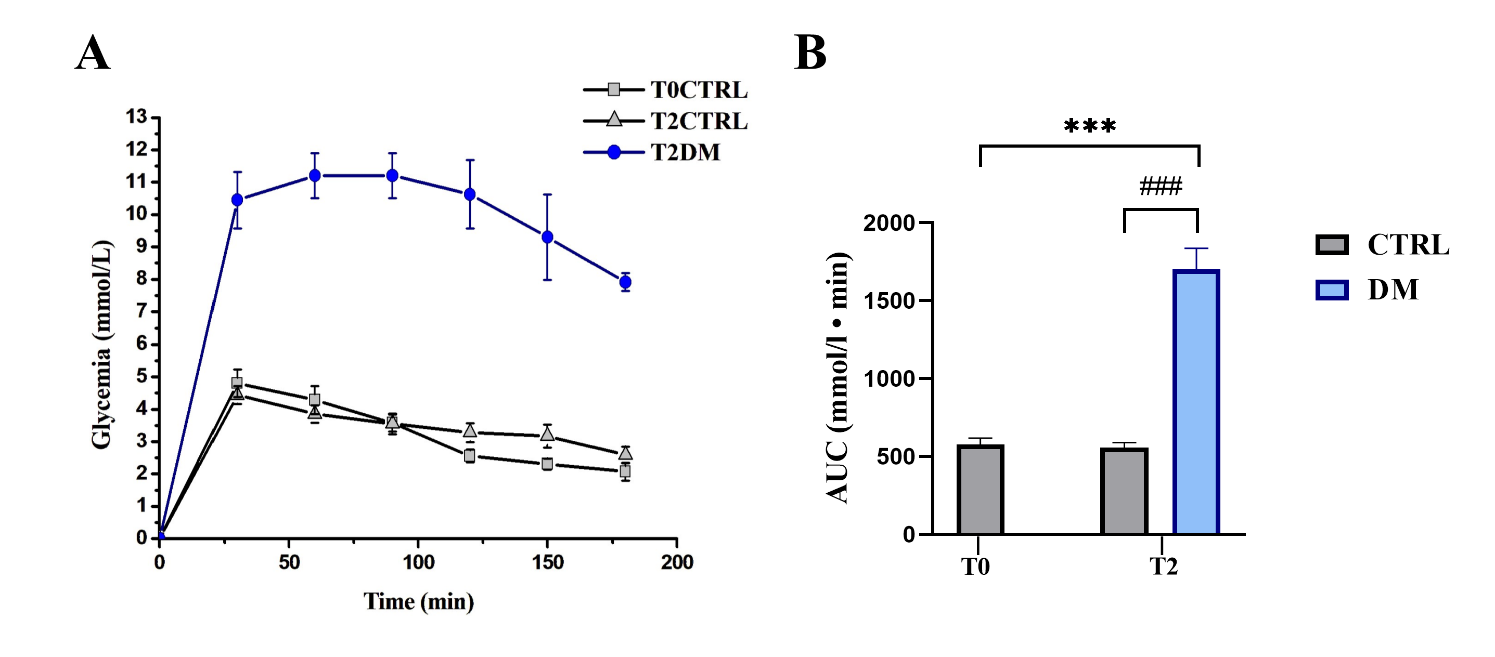


**Figure 1. Glucose tolerance test in control (CTRL, grey), and in diabetic (DM, blue) mice: (A)** mean of response glucose curves at T0 in CTRL mice (square) and at T2 in CTRL (triangle) and DM (circle) mice, and **(B)** mean of Area under the curve (AUC) of Oral glucose test (OGTT) at T0 and T2. Statistical significance (One-Way ANOVA followed by Bonferroni *post-hoc* test): * vs T0, # vs T2 CTRL.For all symbols reported p < 0.05 (*, #); p < 0.01 (**, ##); p < 0.001 (***, ###).

The direct comparison between all CTRL and DM mice is shown in Figure 2 and was described in detail in the main text (Results section 3.2).


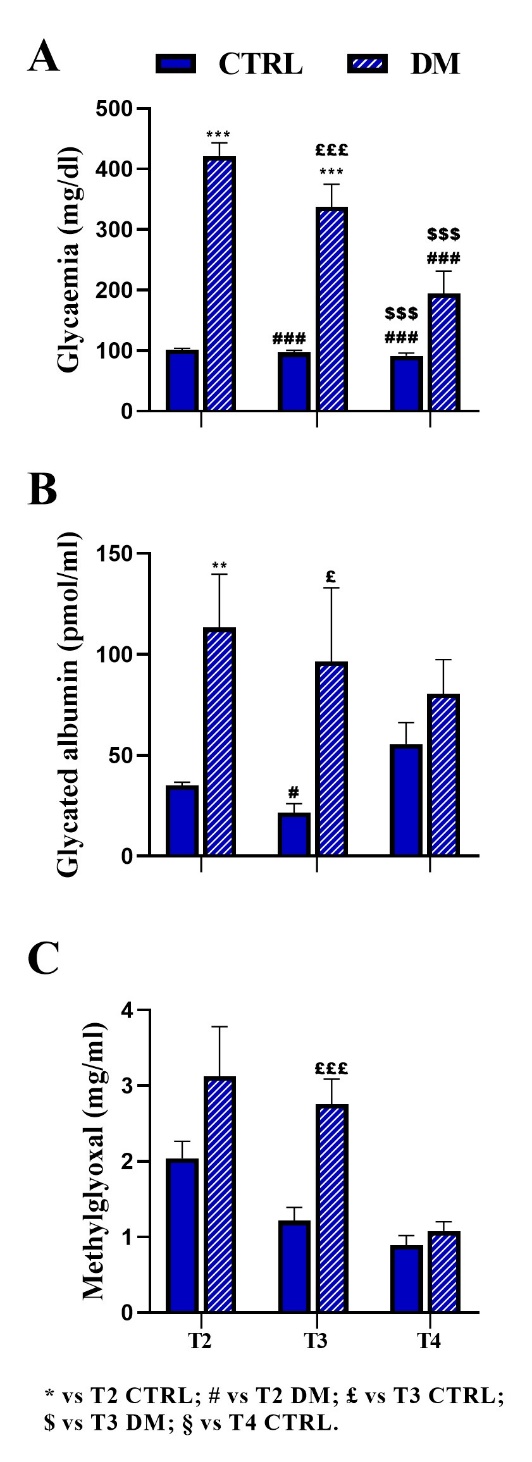


**Figure 2. Comparison between control (CTRL) and diabetic mice (DM) at different experimental timepoints.** Fasting glycemia (**A**), Glycated albumin (**B**), and Methylglyoxal (**C**) levels were assessed in CTRL and DM mice at T2, T3, and T4. The values are reported as mean ± Standard Error of the Mean (SEM). Statistical significance (One-Way ANOVA followed by Bonferroni *post-hoc* test): * vs T2 CTRL, # vs T2 DM, £ vs T3 CTRL, $ vs T3 DM, § vs T4 CTRL. For all symbols reported p < 0.05 (*, #, £, $); p < 0.01 (**, ##, ££, $$); p < 0.001 (***, ###, £££, $$$).
